# The ventro-medial prefrontal cortex: a major link between the autonomic nervous system, regulation of emotion, and stress reactivity?

**DOI:** 10.1186/1751-0759-2-21

**Published:** 2008-11-05

**Authors:** Alexander Hänsel, Roland von Känel

**Affiliations:** 1 Department of General Internal Medicine, Division of Psychosomatic Medicine, University Hospital Berne, Berne, Switzerland

## Abstract

Recent progress in neuroscience revealed diverse regions of the CNS which moderate autonomic and affective responses. The ventro-medial prefrontal cortex (vmPFC) plays a key role in these regulations. There is evidence that vmPFC activity is associated with cardiovascular changes during a motor task that are mediated by parasympathetic activity. Moreover, vmPFC activity makes important contributions to regulations of affective and stressful situations.

This review selectively summarizes literature in which vmPFC activation was studied in healthy subjects as well as in patients with affective disorders. The reviewed literature suggests that vmPFC activity plays a pivotal role in biopsychosocial processes of disease. Activity in the vmPFC might link affective disorders, stressful environmental conditions, and immune function.

## Introduction

Since Walter Cannon described the behavioral and physiologic sequels of the fight-flight response in cats, research has come a long way by studying the many physiologic consequences of acute and chronic stress conditions in humans [1,2]. The fight-flight response is essentially adaptive and includes a change of activity in the autonomic nervous system. In the long run, however, increases in the sympathetic and decreases in the parasympathetic nervous system, respectively, may lead to increased cardiovascular morbidity and mortality [3,4].

Cardiovascular morbidity and mortality is affected by mental disorders as major depressions. For instance, patients with a co-morbid major depressive disorder show a poor prognosis after myocardial infarction [5]. Interestingly, depressive disorders are associated with disturbances in autonomic nervous system activity measured by heart rate variability [6]. Moreover, other mental disorders such as panic disorder or social phobia also show altered functions of the autonomic nervous system [7-9].

The question about which brain areas are related to mental disorders still needs to be answered. However, some insight has been gained in brain areas responsible for changed activity in the autonomic nervous system. There is also a growing literature on neuroscientific mechanisms in emotion regulation. This evidence may help to better understand how mental processes are related to alterations in peripheral physiology and to explain how mental disorders might influence the course of somatic disease.

This review aims to focus mainly on the ventro-medial prefrontal cortex (vmPFC). This area is considered a core region of the emotional brain [10]. Damage of the vmPFC involves symptoms of blunted emotional experience and responses, defective social decision making, impaired goal directed behavior, and lack of insight [11]. It is of particular importance to this review that the vmPFC is related to sympathetic and parasympathetic activity and stress reactivity.

The review is divided into three parts. Firstly, we summarize the influence of the ventro-medial prefrontal cortex (vmPFC) on cardiovascular regulations. Secondly, data on how the vmPFC contributes to the regulation of emotional and cognitive processes is presented. Thirdly, we discuss the contributions of vmPFC activity to stress reactivity.

## vmPFC and cardiovascular modulation

Animal research suggests an important role of vmPFC activity for cardiovascular modulation. If the vmPFC is stimulated in rats, mean arterial pressure decreases and sympathetic tone is inhibited [12,13]. In addition, glutamatergic synapses in the vmPFC modulate the parasympathetic component of baroreflex in rats [14]. Inactivation of the vmPFC, however, withdraws parasympathetic input to the baroreflex while sympathetic input is maintained [15].

In humans the vmPFC is primarily active when subjects are in a restful, but mentally alert state as well as during sleep [16,17]. A recent investigation studied cardiovascular changes and brain activity. Functional magnetic resonance imaging (fMRI) revealed that during an isometric handgrip task performed with low intensity relatively lower activation of the vmPFC correlated with relatively higher heart rate and mean arterial pressure during the task [18]. Since sympathetic activity remained unchanged, the hemodynamic changes evoked by this mechanic-effortful task were discussed mainly to reflect vagal withdrawal. Consequently, decreased vmPFC activity could be assumed to be associated with a reduction in vagal activity.

It is important to emphasize that vagal activity does not solely rely on vmPFC activity. Gianaros et al. measured vagal activity and PET activity during a graded memory task [19]. This study revealed that regional cerebral blood flow covaried positively with vagal activity not only in the right vmPFC but also in the left insula as well as the left amygdala-hippocampal complex and covaried negatively with the right cerebellum.

However, it is unclear whether vmPFC activity is involved in every vagal modulation. Using a novel method to sample efferent cardiovagal outflow and involved central command, Napadow et al. combined heart rate variability and functional magnetic resonance imaging (fMRI) during a grip task [20]. As a result, the fluctuation in vagal activity measured by high frequency (HF) power was correlated with activity in the brainstem, mesencephalon, and diencephalon, as well as in other subcortical and cortical brain regions. A positive correlation of HF power was found in the left hypothalamus, left amygdala, right anterior hippocampus, and right dorsomedial and dorsolateral prefrontal cortex. Negative correlations were also found in regions different from the vmPFC.

Thus, contextual conditions of a given task may define involvement of vmPFC activity in vagal modulation.

## vmPFC and regulation of emotion

Damasio et al. previously performed neuropsychologic testing in patients with damaged vmPFC and found altered psychophysiologic response and changes in emotional experience to emotional but not to neutral stimuli [21]. These findings lend ground to the somatic marker hypothesis which assumes that the vmPFC uses emotion-based biasing signals generated by the body (the somatic marker) when a person appraises different response options [22]. However, a current critical review of the somatic marker hypothesis concluded that there is a need for additional empirical data to support the somatic marker theory [23].

Hilz et al. studied the role of the vmPFC in cardiovascular sequelae while watching pictures with a pleasant emotional content [24]. The group compared healthy volunteers to patients with a loss of their vmPFC area. Lesions originated mostly due to head injury, arteriovenous malformation, and tumors. The presentation of emotionally pleasant slides to healthy subjects generated a significant decrease in heart rate, while blood pressure remained unchanged. Interestingly, the presentation of the same slides to patients with lesions to the left vmPFC was followed by no significant change in heart rate and blood pressure. Patients with loss of the right vmPFC, however, responded differently to the other groups in so far as they showed significant increases in heart rate and blood pressure after the pleasant slides. This difference in cardiovascular responses to emotional stimuli in patients with right-sided and left-sided vmPFC damage points to a possible hemispheric specialization. It seems as if the left vmPFC predominantly governs parasympathetic activation, while the right vmPFC primarily inhibits sympathetic nervous system activity.

The results of different cardiovascular modulations by the left and right hemispheres by Hilz et al. concur with cardiovascular findings following temporary hemispheric inactivation. In this case, pharmacological inactivation of the right hemisphere yields a larger and faster increase of heart rate than inactivation of the left hemisphere [25].

The role of the vmPFC was further investigated in an experiment in which subjects were instructed to regulate their affective response reaction to emotionally negative or neutral pictures [26]. Regulation was defined as volitionally enhancing, suppressing, or maintaining the affective response to the pictures. The magnitude of amygdala activity subsequently decreased from the conditions of affectively enhancing, maintaining, and suppressing responses. Decreased amygdala activity correlated with increased activity in the vmPFC during affective suppression. This finding suggests that higher vmPFC activity occurs in suppression towards a negative emotional signal and might dampen amygdale activity.

The evidence that depressive disorders impair the regulatory function of the vmPFC on amygdala activity is of interest for mental diseases. In a study by Johnstone et al. patients with major depressive disorders (MDD) viewed emotionally positive and negative pictures taken from the International Affective Picture System (IAPS) [27]. Shortly after the start of the presentation, participants were instructed to increase or decrease their emotional response using reappraisal. Compared to healthy individuals who, as expected, had a negatively correlated activity of amygdala and vmPFC, depressed patients showed a positive association between the two regions. Another group investigated vmPFC activation after emotional stimulation in depressed patients [28]. This study used stimuli of happy and sad autobiographical memory scripts and congruent facial expressions. Results of vmPFC activity differed in terms of responses to the emotional stimuli and revealed a reversed pattern. Depressed individuals responded to happy stimuli with an increase of vmPFC activity while healthy subjects showed a decreased response. This reversed pattern was also documented for sad stimuli: depressed subjects showed decreased vmPFC activity while healthy subjects responded with increased activity. Interestingly, depressed individuals subjectively reported an increase in happy mood to happy stimuli but, unlike the healthy individuals, did not demonstrate an increased autonomic response to happy stimuli measured by skin conductance response.

## vmPFC and stress reactivity

Studies of animals and humans assume an important role of the vmPFC in the decision as to whether an environmental signal is perceived as a stressor. Studies of rats showed that controllability of experienced stress is associated with vmPFC activity [29]. In other words, stressful situations primarily affect the serotonin-rich area of the dorsal raphe nucleus (DNR) and DNR stimulation depends on how aversive a stimulus is perceived. The study also showed that – if the neurons of the DNR are blocked experimentally by a serotoninergic agonist – the rats' behavioral freezing response to uncontrollable stress is also blocked [30,31]. The activation of the connection between the vmPFC and the DNR seems to block DNR neurons, because the DNR no longer experiences suppressed activity if the vmPFC is experimentally inactivated [29].

Humans can perceive an environmental context as more stressful through a heightened fear response, which is of clinical importance in anxiety disorders and post-traumatic stress disorders. In these conditions the vmPFC seems to be involved in the decision about how threatening a signal is perceived. Phelps et al. explored neural mechanisms of the extinction of previously fear-conditioned stimuli in humans. They found that activity of the vmPFC is primarily related to the retention of extinction learning [32]. A similar result was found by Milad et al. who reported a significant activation in the vmPFC in response to the extinction of previously conditioned stimuli [33]. The group also reported a correlation between cortical thickness of the vmPFC region and extinction recall [34]. To summarize, the studies by Phelps et al. and Milad et al. suggest that the activity in the vmPFC area is related to the process of eliminating previously learned associations of fearful conditioned signals. Thus, vmPFC activity seems to be involved in the process of resilience, the dynamic process of positive behavioral adaptation after encountering significant adversity or trauma [35].

Clinical situations with reduced resilience are found in subjects suffering from anxiety disorders. A recent meta-analysis reviewed studies of functional neuroimaging in patients with post-traumatic stress disorder (PTSD), social anxiety disorder, and specific phobia [36]. Overall, PTSD patients show less activity in the vmPFC than healthy subjects when experiencing or regulating emotions. Their vmPFC activity is also decreased when they are exposed to reminders of traumatic events [37].

## Summary and discussion

The main findings of this review are the following: 1) Research suggests that the vmPFC area, especially of the right hemisphere, plays an important role in regulating parasympathetic activity; 2) vmPFC activation is associated with successful suppression of affective responses to a negative emotional signal; 3) depressive disorders are associated with a dysfunctional interplay between vmPFC and amygdala activity, whereby the physiological inhibition of the amygdala by enhanced vmPFC activity is eliminated; 4) the vmPFC becomes activated if a situation is perceived as controllable and if an organism has learned to delete a previously fear-conditioned signal. The different results of activation and inhibition of vmPFC activity are summarized in Table 1.

**Table 1:** Functional states associated with vmPFC-activity

**Increased activity:**	**Decreased activity:**
Rest, sleep	Low intense motor activity

Elevated parasympathetic activity	Posttraumatic stress disorder

Lowered sympathetic activity	Depressive Disorder

Suppression of negative emotional signal	

Extinction of previously fearfully conditioned stimuli	

Controllability over stressful situation	

The above synthesis of the literature must be seen within the limits of this review, which was selective and not intended to be exhaustive. The vmPFC is only one region of the central autonomic network that includes a series of prefrontal and limbic structures [38]. Therefore, one might argue that focusing on the role of the vmPFC alone oversimplifies the complex central regulation of parasympathetic activity. In addition, the bulk of the reviewed literature did not investigate heart rate variability as a measure of autonomic modulation of the heart and conclusions on parasympathetic outflow to the heart as affected by the vmPFC are notional.

If vmPFC activity is relevant to vagal nerve function, the vmPFC might play a key role in mediating the relationship of affective disorders and stressful psychosocial factors with changed activity in the autonomic nervous system. There is evidence in rodents that chronic stress leads to volume loss, dentritic atrophy and decreased left to right inhibition in the medial prefrontal cortex that is followed by disturbance of the autonomic nervous system and behavioral dysfunction [39]. Therefore, it seems promising to record autonomic nervous system function by means of e.g. heart rate variability and baroreflex sensitivity while also studying brain activity by functional brain imaging studies in future investigations in humans.

## Competing interests  

The authors declare that they have no competing interests.

## Authors' contributions

AH and RvK participated in the writing of the manuscript. Both authors read and approved the final manuscript.
